# Analysis of the Influence of IL-6 and the Activation of the Jak/Stat3 Pathway in Fibromyalgia

**DOI:** 10.3390/biomedicines11030792

**Published:** 2023-03-06

**Authors:** Ylenia Marino, Alessia Arangia, Marika Cordaro, Rosalba Siracusa, Ramona D’Amico, Daniela Impellizzeri, Rosalia Cupi, Alessio Filippo Peritore, Enrico Gugliandolo, Roberta Fusco, Salvatore Cuzzocrea, Rosanna Di Paola

**Affiliations:** 1 Department of Chemical, Biological, Pharmaceutical and Environmental Sciences, University of Messina, Viale Ferdinando Stagno D’Alcontres, n 31, 98166 Messina, Italy; 2 Department of Biomedical, Dental and Morphological and Functional Imaging, University of Messina, Via Consolare Valeria, 98125 Messina, Italy; 3 Department of Veterinary Sciences, University of Messina, Viale Annunzita, 98168 Messina, Italy

**Keywords:** fibromyalgia, chronic pain, cytokines

## Abstract

Background: Fibromyalgia is a medical condition that affects a small percentage of the population, with no known effective treatment. There is evidence to suggest that inflammation is a key factor in the nerve sensitization that characterizes the disorder. Therefore, this paper concentrates on the role of IL-6 in fibromyalgia and the related pain-like symptoms. Methods: This work aimed to evaluate Sprague–Dawley rats, which were injected for three consecutive days with 1 mg/kg of reserpine; IL-6-R Ab was intraperitoneally injected at 1.5 mg/kg seven days after the first reserpine injection. Behavioral analyses were conducted at the beginning of the experiment and at seven and twenty-one days from the first reserpine injection. At this timepoint, the animals were sacrificed, and tissues were collected for molecular and histological analysis. Results: Our data showed the analgesic effect of IL-6-R-Ab administration on mechanical allodynia and thermal hyperalgesia. Additionally, the reserpine + IL-6-R-Ab group showed a reduced expression of the pain-related mediators cFOS and NFG and reduced levels of pro-inflammatory cytokines (TNF-α, IL-1β and IL-6) and chemokines (Cxcl5, Cxcl10 and Cx3cl1). From the molecular point of view, the IL-6-R-Ab administration reduced the gp130 phosphorylation and the activation of the Jak/STAT3 pathway. Additionally, the IL-6-R Ab reduced the activation of neuroinflammatory cells. Conclusions: Our study showed that IL-6 plays a crucial role in fibromyalgia by triggering the Jak/STAT3 pathway, leading to an increase in chemokine levels and activating glial cells.

## 1. Introduction

Fibromyalgia is a chronic condition characterized by pervasive pain, fatigue and depression [1,2]. It is widespread: currently, it affects the 6% of people [3,4,5]. Its main characteristic is the damaged transduction of nociceptive signaling. This disfunction leads to hypersensitivity to non-noxious stimuli [6,7].

Although peripheral sensitization certainly contributes to the sensitization of the nociceptive system and, thereby, to inflammatory-pain hypersensitivity at inflamed sites (primary hyperalgesia) [8], it nevertheless represents a form of pain elicited by the activation of nociceptors, albeit one with a lower threshold due to the increased peripheral transduction sensitivity, and generally involves ongoing peripheral pathology [9]. Peripheral sensitization appears to play a major role in altered heat, but not mechanical sensitivity, which is a major feature of central sensitization [10,11]. The etiology of fibromyalgia is still unclear: if central sensitization is considered to be the main mechanism involved, then many other genetic, immunological and hormonal factors may play an important role [12]. In the past, fibromyalgia was considered a non-inflammatory disorder [13,14]; currently, many pro-inflammatory pathways and mediators are known to be activated in fibromyalgia patients [15,16,17,18,19]. Several clinical and experimental reports displayed the key role of inflammation in fibromyalgia [20,21,22,23]. The increased release of pro inflammatory cytokines has been detected in suffering animals, and their overexpression is correlated with the development of pain-like behaviors [24,25,26]. Thus, neuropathic pain is characterized by a pro-inflammatory microenvironment [27]. These mediators are secreted by immune/inflammatory cells [28,29,30,31] and include interleukin-6 (IL-6), interleukin-1 beta (IL-1β), tumor necrosis factor-α (TNF), interleukin-10 (IL-10), monocyte chemoattractant protein 1 (MCP-1), glutamate, nerve-growth factor (NGF) and substance P (SP) [32,33,34]. Among these pro-inflammatory mediators, one of the most thoroughly studied is interleukin-6 (IL-6). Several papers described the relationship between neuropathic pain and IL-6 [35]. It is a pleiotropic cytokine responsible for many biological pathways, including neuropathologies [36,37]. Originally described as a B-stimulatory factor in the production of immunoglobulin [38], IL-6 binds its receptor IL-6-R on target cells [39,40] and activates the signal transducing membrane glycoprotein gp130 [41]. In turn, it dimerizes and activates several intracellular signaling pathways, including mitogen-activated protein kinase/extracellular signal-regulated kinase (MAPK/ERK), the Janus-activated kinase/signal transducer activator of transcription (JAK/STAT) and phosphatidylinositol 3-kinase/protein kinase B (PI3K/Akt) signaling pathways [42,43]. Persistent IL-6 increase is correlated with chronic pain in both patients [44] and experimental animals [45]. Its role has been described in rheumatoid-arthritis-induced pain [46], spinal-cord-injury induced pain [47], cancer pain [48], neuropathic pain [49], peripheral nerve injury [50], chemotherapy-induced peripheral neuropathy [51] and inflammatory pain [52]. In contrast with other sources of pathological pain, such as cancer and neuropathic pain, fibromyalgia syndrome is a chronic painful condition, which is characterized by widespread pain mainly perceived in deep somatic tissues, i.e., in the muscles and joints. The definition is based on the American College of Rheumatology (ACR)’s classification scheme [53]. Fibromyalgia (FM) is also characterized by abnormal pain sensitivity and frequent additional comorbidities, such as sleep disturbances and affective disorders [53]. In contrast to classic neuropathic pain syndromes, the general perception of fibromyalgia is that in this disease, nerve lesions are not demonstrable [54,55]. In particular, increased expression levels of IL-6, IL-6-R, and gp130 have been found in dorsal-root ganglia (DRG) and spinal-cord tissues from suffering animals. Moreover, experimental reports displayed that IL-6 injection induces thermal hyperalgesia and mechanical allodynia. Additionally, it enhances translations in sensory neurons and links to nociceptive plasticity [56,57], contributing to central sensitization [58,59,60]. These previous data suggest the key role of IL-6 in pathological pain. To the best of our knowledge, there is no complete description of IL-6’s role in the chronic pain related to fibromyalgia, although some authors previously approached this topic [61,62]. Thus, this paper focuses on the role of IL-6 fibromyalgia and associated pain-like behaviors. To perform this analysis, we employed an animal model of reserpine-induced fibromyalgia, a biogenic amine depletory. In particular, we employed a validated model comprising three reserpine administrations [63], although recently other authors reported higher numbers of reserpine injections [64]. In our model, long-lasting widespread nociceptive hypersensitivities were exhibited in rats [65,66]. It is noteworthy that the time course of pain-related behaviors was in parallel with the decrease in monoamine neurotransmitters after the reserpine treatment [66]. Indeed, reserpine promotes the appearance of the inflammatory process, including the expression of cytokines and growth factors by macrophages and mesangial cells (IL-1b, IGF-1 and TNF-α) [67]. Thus, monoamine depletion appears to cause nociceptive hypersensitivity in a rat-reserpine-induced pain model, which could be useful in the study of the pathological mechanisms of chronic, widespread pain, including fibromyalgia.

## 2. Materials and Methods

### 2.1. Animals

Sprague–Dawley male rats (200–220 g) were employed for this research. Food and water were managed ad libitum. The University of Messina Review Board for animal care approved the study (212/2021-PR). All experiments followed the USA (Animal Welfare Assurance No. A5594-01), European (EU Directive 2010/63) and Italian (D. Lgs 2014/26) guidelines.

### 2.2. Induction of Fibromyalgia

Reserpine was administered by 3 subcutaneous injections of 1 mg/kg for 3 consecutive days [26,63,65]. Reserpine was dissolved in distilled water with 0.5% acetic acid (vehicle). Controls received the same volume as vehicle, but no reserpine was injected.

### 2.3. IL-6 Ab Administration

The IL-6-R Ab (R&D systems) was dissolved in PBS and intraperitoneally injected at 1.5 mg/kg seven days after the first reserpine injection [47].

### 2.4. Experimental Groups

Rats were randomly divided into several groups (n = 20 for each).

Control: animals were subcutaneously injected with vehicle instead of reserpine and treated with PBS seven days after the first reserpine injection.

Control + IL-6-R Ab: animals were subcutaneously injected with saline instead of reserpine and treated with IL-6-R Ab seven days after the first reserpine injection.

Reserpine: animals were subcutaneously injected with reserpine as described above.

Reserpine + IL-6-R Ab: animals were subcutaneously injected with reserpine as described above and treated with IL-6-R Ab seven days after the first reserpine injection.

Twenty-one days after reserpine injection, behavioral analyses were conducted, animals were sacrificed by isoflurane overdose and samples from L4–L6 area of spinal cord were collected for molecular analysis.

### 2.5. Behavioral Analyses

Behavioral analyses were performed on day 0 to obtain initial data, on day 7 before the IL-6-R-Ab administration to verify the reserpine-induced fibromyalgia and, subsequently, after 21 days from the beginning of the experiment to assess the effect of IL-6-R-Ab administration on behavioral changes. We decided to perform these analyses on these days based on previously published data on IL-6-R Ab [47] and on the experimental model [68].

#### 2.5.1. Von Frey Hair Test

Mechanical allodynia was evaluated at 0, 7 and 21 days from the first reserpine injection, using a dynamic plantar von Frey hair esthesiometer (Bio-EVF4; Bioseb, Vitrolles, France) [69]. The device includes a force transducer with a plastic tip. The tip was applied to the plantar area and a rising, upward force was exerted. The withdrawal threshold was defined as the force, expressed in grams, at which the mouse removed its paw.

#### 2.5.2. Hot-Plate Test

At 0, 7 and 21 days from the first reserpine injection, the hot-plate test was performed. The hot-plate latency was calculated using a metal surface maintained at 53.6 °C (Ugo Basile, Milan, Italy). Each rat was monitored, and the licking of a hind paw was set as the end point [70]. Maximal latency accepted was 45 s. Regarding thermal hyperalgesia, the hot-plate test is advantageous over tail flicking/tail withdrawal because it gives the opportunity to test thermal sensitivity unconfounded by stress-induced analgesia associated with restraint.

#### 2.5.3. Tail-Flick Warm-Water Test

At 0, 7 and 21 days from the first reserpine injection, the tail-flick warm-water test was performed to evaluate pain threshold (IITC Life Science). Each rat’s tail was immersed in warm water (50 ± 0.5 °C) and the time between tail input and retraction was recorded. A maximum tail-flick latency of 10 s was employed to minimize tissue damage [71].

### 2.6. Quantitative Real-Time PCR

Total RNA from spinal-cord tissue was extracted according with the manufacturer’s instructions (Qiagen, Milan, Italy). The RNA was quantified using a Nanodrop spectrometer and cDNA was obtained using iScriptTM cDNA Synthesis Kit (Bio-Rad, Milano, Italy). according to manufacturer’s protocols [72]. Real-time PCR analysis was performed by the SYBR Green method using the QuantiTect Primer Assay (Qiagen) with b-actin as internal control [73]. Real-time PCR was performed using a Bio-Rad CFX Real-Time PCR ((Bio-Rad, Milano, Italy) Detection System [74].

### 2.7. Western-Blot Analysis

Western-blot analysis was performed on lumbar-spinal-cord tissues. Samples were prepared as described previously [75]. Briefly, tissues were suspended in buffer A (0.2 mM phenylmethylsulfonyl fluoride, 0.15 mM pepstatin A, 20 mM leupeptin and 1 mM sodium orthovanadate), homogenized for 2 min and centrifuged at 10,000× *g* for 10 min at 4 °C. Supernatants represented the cytosolic fraction. The pellets, containing nuclei, were re-suspended in buffer B (150 mM NaCl, 1% Triton X-100, 1 mM EGTA, 1 mM EDTA, 10 mM Tris–HCl pH 7.4, 0.2 mM phenylmethylsulfonyl fluoride, 20 mM leupeptin and 0.2 mM sodium orthovanadate). After centrifugation for 30 min at 15,000× *g* at 4 °C, the supernatants contained nuclear proteins. Equal amounts of protein were separated on SDS-PAGE gel and transferred to nitrocellulose membrane [76]. The membranes were probed with specific antibodies: (sc-377573) p-gp130 antibody, (A94191) p-Jak1 antibody, (ab32143) p-STAT3 antibody, (sc-32300) NGF antibody, (sc-166940) cFOS antibody in 1x PBS, 5% *w*/*v* non-fat dried milk and 0.1% Tween-20 at 4 °C, overnight [77]. Filters were incubated with peroxidase-conjugated bovine anti-mouse IgG secondary antibody or peroxidase-conjugated goat anti-rabbit IgG (1:5000, Jackson ImmunoResearch, West Grove, PA, USA) for 1 h at room temperature. To ensure amounts of proteins were equal, blots also were probed with an antibody against the b-actin protein (Santa Cruz Biotechnology). Signals were examined with an enhanced chemiluminescence (ECL) detection-system reagent (Thermo Fisher, Waltham, MA, USA) [78]. The relative expression of the protein bands was quantified by densitometry with BIORAD ChemiDocTM XRS+ software and standardized to b-actin. Each blot was stripped with glycine 2% and re-incubated several times to optimize the detection of the proteins and to visualize other proteins, minimizing the number of gels and transfers.

### 2.8. Immunohistochemical Analysis

Immunohistochemical analysis was performed on sections (7 μm) of spinal cord, as previously described [79]. Briefly, tissues were fixed in 10% (*w*/*v*) PBS-buffered formaldehyde and embedded in paraffin. Seven-micrometer sections were prepared from tissues. After deparaffinization, endogenous peroxidase was quenched with 0.3% (*v*/*v*) hydrogen peroxide in 60% (*v*/*v*) water for 30 min. The slides were permeabilized with 0.1% (*w*/*v*) Triton X-100 in PBS for 20 min. Tissue sections were incubated in 2% (*v*/*v*) normal goat serum in PBS to block non-specific binding. The sections were incubated overnight with primary antibodies: anti-GFAP (SCB, #sc33673) and anti-IBA-1 (Thermo Fisher Scientific, Milan, Italy) antibodies. Slides were then washed with PBS and incubated with a secondary antibody. Specific labeling was identified with an avidin–biotin-peroxidase complex and a biotin-conjugated goat anti-rabbit immunoglobulin G (Vector Lab, Milan, Italy). Five histological sections were evaluated for each animal. Cells were enumerated by counting five high-power fields (40×) per section using Leica DM6 microscope (Leica Microsystems, Milan, Italy). Reactive glia cells were considered as highly ramified with hypertrophic processes [80,81].

### 2.9. ELISA Analysis

The concentrations of TNF-α, IL-1β and IL-6 were measured. Briefly, spinal-cord tissues were homogenized in 1 mL PBS with 10 μL of protease inhibitor at low speed. The samples were centrifuged at 14,000× *g* at 4 °C for 15 min; supernatants were employed, using respective ELISA kits according to the manufacturer’s protocol and analyzed using a microplate reader [82].

### 2.10. Statistical Analysis

All values in the figures and text are expressed as mean ± standard error of the mean (SEM) of N = 5 number of animals. The results of the behavioral analysis were analyzed by two-way ANOVA, while all other results were analyzed by one-way ANOVA. Both analyses were followed by a Bonferroni post hoc test for multiple comparisons. A *p*-value < 0.05 was considered significant; * *p* < 0.05 vs. control; # *p* < 0.05 vs. reserpine; ** *p* < 0.01 vs. control; ## *p* < 0.01 vs. reserpine; *** *p* < 0.001 vs. control; ### *p* < 0.001 vs. reserpine.

## 3. Results

### 3.1. Experimental Timeline

**Figure 1:**
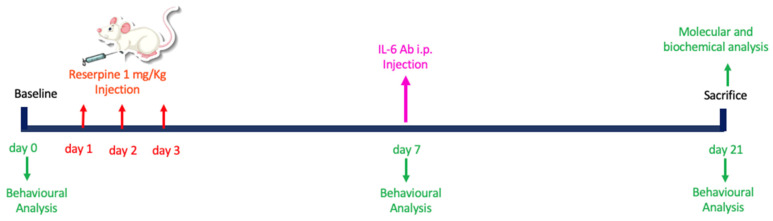
Timeline of the experiment.

In order to investigate the effects of IL-6-R Ab on fibromyalgia, the rats were subcutaneously administered reserpine for three consecutive days and were intraperitoneally injected at seven days after the first reserpine injection with IL-6-R Ab 1.5 mg/kg (Figure 1).

### 3.2. Analysis of Pain-like Behaviours

**Figure 2:**
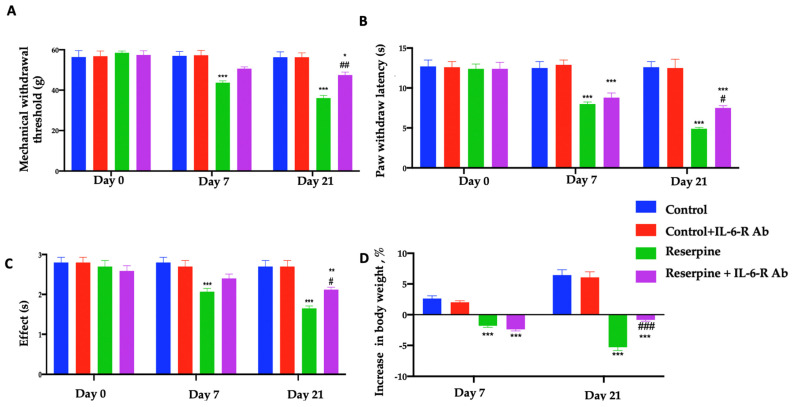
Effects of IL-6-R Ab on behavioral alterations: (**A**) von Frey hair test, (**B**) hot-plate test, (**C**) tail-flick warm-water test, (**D**) weight change.

Seven days after the first reserpine injection, the animals displayed increased sensitivity to mechanical (Figure 2A, *** *p* < 0.001 vs. control) and thermal (Figure 2B,C, *** *p* < 0.001 vs. control) stimuli compared to the control and control + IL-6-R Ab groups. No statistical differences were found between the control and control + IL-6-R Ab groups. This hypersensitivity strongly increased 21 days from the first reserpine injection (*** *p* < 0.001 vs. control), while the animals administered IL-6-R Ab at the same timepoint showed reduced hypersensitivity to von Frey hair (Figure 2A, ## *p* < 0.01 vs. reserpine, * *p* < 0.05 vs. control), hot-plate (Figure 2B, # *p* < 0.05 vs. reserpine, *** *p* < 0.001 vs. control) and tail-flick warm-water (Figure 2C, # *p* < 0.05 vs. reserpine, ** *p* < 0.01 vs. control) tests. The analgesic effect of the IL-6-R-Ab administration on the long-term fibromyalgia-induced changes in the nociceptive pathways persisted. One of the characteristic hallmarks of suffering animals is the reduction in body weight. The control and control + IL-6-R Ab rats gained in body weight during the experiment and no differences were detected between them (Figure 2D). Fibromialgia induced a reduction in body weight in the rats from the reserpine group (*** *p* < 0.001 vs. control). The body weights of rats in the reserpine + IL-6-R-Ab group increased after the IL-6-R-Ab administration compared with those in the reserpine group (*** *p* < 0.001 vs. control, ### *p* < 0.001 vs. reserpine), suggesting that the IL-6-R-Ab-treated fibromyalgia rats did not experience chronic pain (Figure 2D).

### 3.3. Analysis of Pain-Related Mediators

**Figure 3:**
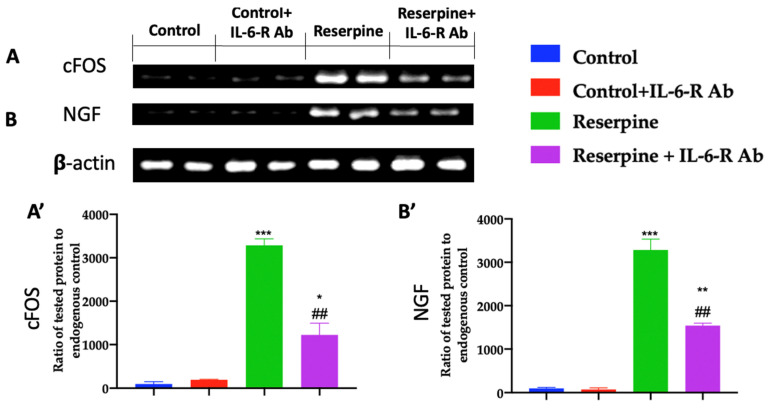
Effects of IL-6-R Ab on pain-related mediators: Western-blot analysis of: (**A**,**A’**) cFOS and (**B**,**B’**) NGF expressions.

To evaluate the nociceptive pathways, Western-blot analyses were performed 21 days from the first reserpine injection. The spinal-cord tissues harvested from the reserpine group showed increased cFOS (Figure 3A,A’, *** *p* < 0.001 vs. control) and NGF expression (Figure 3B,B’, *** *p* < 0.001 vs. control) compared to the control and control + IL-6-R-Ab rats. The animals from the reserpine + IL-6-R-Ab group showed a reduction in both nociceptive mediators in the lumbar-spinal-cord tissues (Figure 3A, ## *p* < 0.01 vs. reserpine, * *p* < 0.05 vs. control and Figure 3B, ## *p* < 0.01 vs. reserpine, ** *p* < 0.01 vs. control).

### 3.4. Analysis of Pro-Inflammatory Mediators

**Figure 4:**
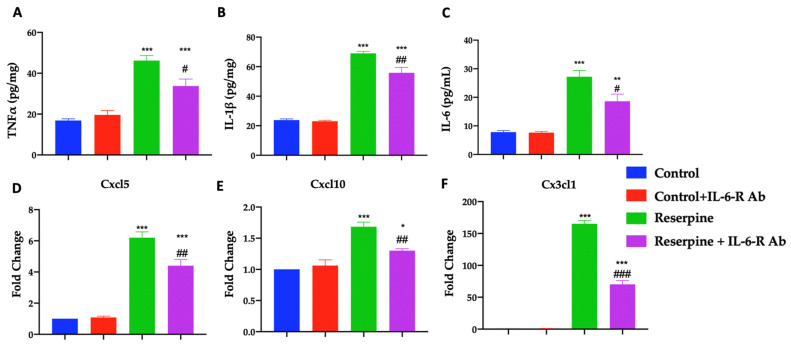
Effects of IL-6-R Ab on pro-inflammatory mediators: ELISA analysis of: (**A**) TNF-α, (**B**) IL-1β and (**C**) IL-6 expressions; RT-PCR analysis of: (**D**) Cxcl5, (**E**) Cxcl10 and (**F**) Cx3cl1 expression.

The expressions of proinflammatory cytokines and chemokines were assessed in the lumbar region of the spinal cords of the experimental animals 21 days from the first reserpine injection. Increased expressions of TNF-α (Figure 4A, *** *p* < 0.001 vs. control), IL-1β (Figure 4B, *** *p* < 0.001 vs. control) and IL-6 (Figure 4C, *** *p* < 0.001 vs. control) were found in the samples from the reserpine group compared to the control and control + IL-6-R-Ab rats. The IL-6-R-Ab administration strongly reduced these levels (Figure 4A, # *p* < 0.05 vs. reserpine, *** *p* < 0.001 vs. control Figure 4B, # *p* < 0.05 vs. reserpine, ** *p* < 0.01 vs. control and Figure 4C, # *p* < 0.05 vs. reserpine, ** *p* < 0.01 vs. control). The RT-PCR analysis was performed to evaluate the chemokine expression. Increased expressions of Cxcl5 (Figure 4D, *** *p* < 0.001 vs. control), Cxcl10 (Figure 4E, *** *p* < 0.001 vs. control) and Cxecl1 (Figure 4F, *** *p* < 0.001 vs. control) were found in the lumbar region of the spinal cords of the reserpine group compared to the control and control + IL-6-R-Ab groups. Samples from the reserpine + IL-6-R-Ab group showed reduced levels of Cxcl5 (Figure 4D, ## *p* < 0.01 vs. reserpine, *** *p* < 0.001 vs. control), Cxcl10 (Figure 4E, ## *p* < 0.01 vs. reserpine, * *p* < 0.05 vs. control) and Cx3cl1 (Figure 4F, ### *p* < 0.001 vs. reserpine, *** *p* < 0.001 vs. control).

### 3.5. Analysis of the Jak/Stat Pathway

**Figure 5:**
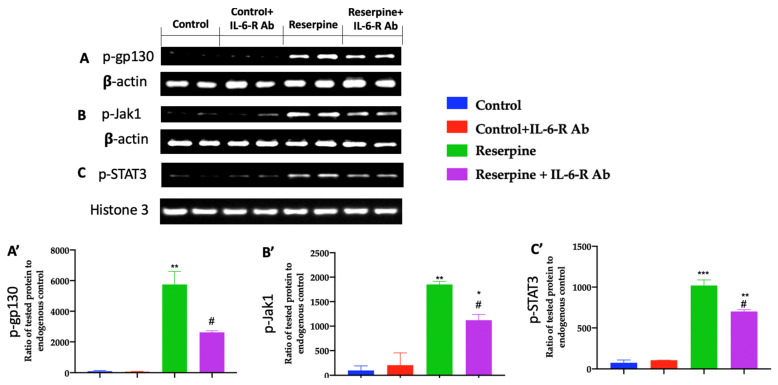
Effect of IL-6-R Ab on pain-related mediators: Western-blot analysis of: (**A**,**A’**) p-gp130, (**B**,**B’**) p-Jak and (**C**,**C’**) p-STAT3 expressions.

A Western-blot analysis was performed 21 days from the first reserpine injection. The analysis showed increased gp130 phosphorylation in the tissues from the reserpine group compared to the control and control + IL-6-R-Ab rats (Figure 5A,A’, ** *p* < 0.01 vs. control). The same trend was found for the Jak phosphorylation (Figure 5B,B’, ** *p* < 0.01 vs. control). The IL-6-R-Ab administration strongly reduced the p-gp130 and p-Jak expression (Figure 5A, # *p* < 0.05 vs. reserpine and Figure 5B, # *p* < 0.05 vs. reserpine, * *p* < 0.05 vs. control). Additionally, the samples from the reserpine group showed increased STAT3 phosphorylation in the nuclear compartment compared to the controls (Figure 5C,C’, *** *p* < 0.001 vs. control). The reserpine + IL-6-R-Ab group showed reduced p-STAT3 expression (Figure 5C, # *p* < 0.05 vs. reserpine, ** *p* < 0.01 vs. control).

### 3.6. Analysis of Glial Activation

**Figure 6:**
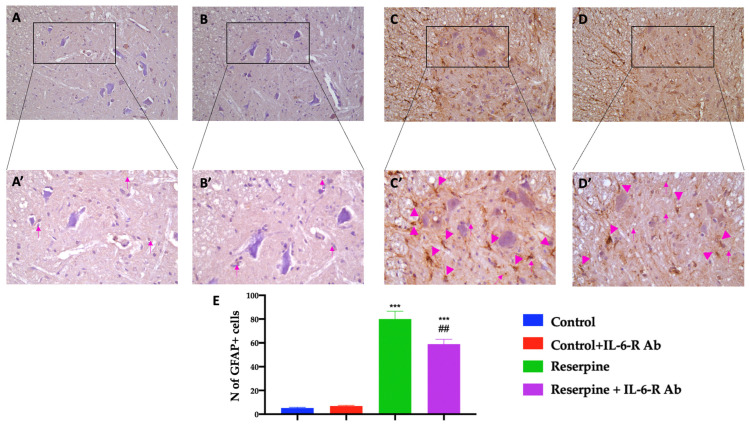
Effects of IL-6-R Ab on astrocyte activation: Immunohistochemical analysis of GFAP expressions: (**A**,**A’**) control, (**B**,**B’**) control + IL-6-R Ab, (**C**,**C’**) reserpine, (**D**,**D’**) reserpine + IL-6-R Ab, graphical quantification of GFAP expressions. Non-reactive glial cells are marked with an arrow, reactive glial cells are marked with an arrowhead, (**E**) N of GFAP + cells.

**Figure 7:**
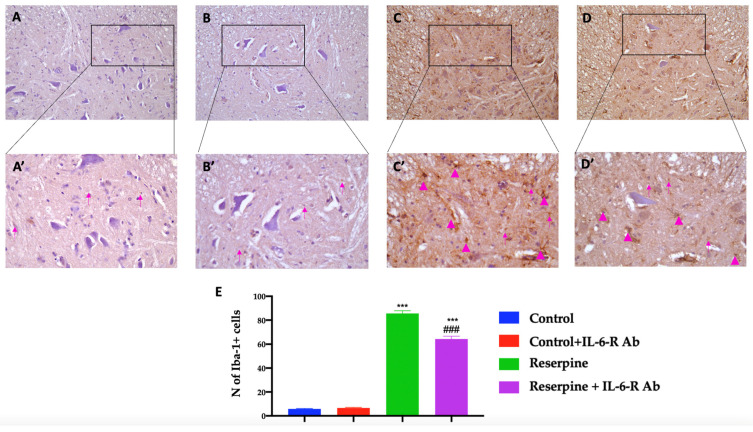
Effects of IL-6-R Ab on microglial activation: Immunohistochemical analysis of Iba-1 expressions: (**A**,**A’**) control, (**B**,**B’**) control + IL-6-R Ab, (**C**,**C’**) reserpine, (**D**,**D’**) reserpine + IL-6-R Ab, graphical quantification of Iba-1 expressions. Non-reactive glial cells are marked with an arrow, reactive glial cells are marked with an arrowhead, (**E**) N of Iba-1 + cells.

An immunohistochemical analysis was performed 21 days from the first reserpine injection on the lumbar spinal-cord tissues. In particular, higher-magnification imaging revealed a small morphology and low level of GFAP and Iba-1 immunoreactivity in the controls (Figure 6A,A’,B,B’ and Figure 7A,A’,B,B’, respectively), while in the reserpine-administered rats, astrocytes and microglia exhibited intense GFAP (Figure 6C,C’, *** *p* < 0.001 vs. control) and Iba-1 (Figure 7C,C’, *** *p* < 0.001 vs. control) immunoreactivity and adopted a highly ramified morphology with hypertrophic processes. The IL-6-R-Ab administration strongly reduced both the GFAP (Figure 6D,D’,E, ## *p* < 0.01 vs. reserpine, *** *p* < 0.001 vs. control) and the Iba-1 (Figure 7D,D’,E, ### *p* < 0.001 vs. reserpine, *** *p* < 0.001 vs. control) expressions, reducing the reactive-glial-cell number.

## 4. Discussion

This study employed a well-established experimental animal model of fibromyalgia to investigate the role of IL-6 in this syndrome.

The leading school of thought views fibromyalgia as a central sensitization syndrome. Nociplastic pain is the recently proposed term to mechanistically explain central sensitization. Accumulating research suggests an alternate explanation: fibromyalgia can be conceptualized as a neuropathic pain syndrome, with dorsal root ganglia (not the brain) as the primary pain source [83].

Firmly in line with the literature, after 3 weeks, the animals exhibited widespread nociceptive hypersensitivities and allodynia [84]. Based on the data presented by Guptarak et al. [47], the IL-6-R Ab was administered seven days after the first reserpine injection. Twenty-one days thereafter, pain-like behaviors were checked. The characteristic hypersensitivity to mechanical and thermal stimuli in fibromyalgia was significantly reduced by the IL-6-R-Ab administration. We also showed that the body-weight increases in the IL-6-R-Ab-administered animals were similar to those shown by the control animals, suggesting that the rats did not suffer chronic pain. Again clearly in line with the literature [68,85], the molecular analysis of the spinal-cord tissues confirmed the increased expression of neuro-sensitizing mediators, such as NGF and c-FOS, in the suffering animals compared to the controls. The IL-6-R-Ab administration reduced the expression of both these mediators, confirming the analgesic effects derived from the neutralizing IL-6-R Ab.

Reserpine is a monoamine depletor that exerts a blockade on the vesicular monoamine transporter for neuronal transmission or storage, promoting dopamine autoxidation and oxidative catabolism by monoamine oxidase [86]. This accelerated mechanism leads to the formation of dopamine quinones and hydrogen peroxide, related to the oxidative-stress process [87]. Bagis et al. [88] demonstrated significantly higher serum levels of pentosidine and malondialdehyde, together with serum superoxide dismutase reduction, in patients with chronic pain compared with normal controls. This generation of advanced glycation end products resulting from increased nitrosative stress activates transcription factor NF-kb, leading to pro-inflammatory gene expression [89]. It includes the expression of cytokines and growth factors by macrophages and mesangial cells (IL-1b, IGF-1, TNF-α). Thus, to evaluate the expression profile of IL-6 in fibromyalgia, further molecular analyses were performed. Increased levels of pro-inflammatory cytokines, such as TNF-α, IL-1β and IL-6 and their mediators, were detected in the suffering animals compared to the controls, confirming the key role of inflammation in fibromyalgia [90,91]. Among these circulating biomarkers, calcitonin gene-related peptide (CGRP) is also dysregulated and may be a reliable parameter to help diagnose this complex syndrome [92], while there were no differences in vascular endothelial growth factor (VEGF) and nitric oxide (NO) levels between patients and controls [93]. The IL-6-R-Ab administration reduced proinflammatory cytokine levels. In particular, the IL-6-R Ab blocked IL-6-related signal transduction.

Reserpine administration by increasing cytokine levels increases the activation of cytokines on the pathways they subtend [67]. Once it is bounded to its receptor, IL-6 recruits gp130 to its receptor complex, which is responsible for Jaks activation and STAT-protein recruitment [94]. The IL-6/IL-6-R complex induces gp130 homodimerization, which in turn activates Jaks and STAT3 downstream proteins. Phosphorylated gp130 provides docking sites for STAT3, which is then phosphorylated by JAKs. The P-STAT3 dimers translocate to the nucleus, where they regulate gene expression binding to specific DNA elements [95]. The IL-6-R-Ab administration reduced gp130 and Jak1 phosphorylation and, in turn, STAT3 phosphorylation. Furthermore, STAT3 has a key role in nociceptive transmission [96,97,98]; in our study, the inhibition of the Jak/STAT3 pathway by the IL-6-R-Ab administration reduced the pain-like behaviors induced by fibromyalgia. Recent studies underline the key role of STAT3 in chronic pain [99]. In particular, the Jak/STAT3 signaling was related to cellular proliferation and astrocyte activation in an animal model of chronic pain [100]. Emerging lines of evidence indicate that peripheral-nerve injury converts resting spinal-cord glia into reactive cells, which are required for the development and maintenance of neuropathic pain. It was found that nerve-injury-induced astrocyte proliferation requires the JAKs/STAT3 pathway.

The rats’ nerve injuries induced marked signal transduction and activation of STAT3 translocation and activation in the dorsal horn astrocytes. Intrathecally administering inhibitors of JAKs and STAT3 3 signaling to rats with nerve injuries reduced the number of proliferating dorsal horn astrocytes and produced recovery from established tactile allodynia, a cardinal symptom of neuropathic pain that is characterized by pain hypersensitivity evoked by innocuous stimuli. Moreover, recovery from tactile allodynia was also produced by the direct suppression of dividing astrocytes by the intrathecal administration of the cell -cycle inhibitor flavopiridol to the rats with injuries. Together, these results imply that the JAKs/STAT3 signaling pathways are critical transducers of astrocyte proliferation and the maintenance of tactile allodynia and may be a therapeutic target for neuropathic pain [101,102]. Strongly in line with previous data [103], the activation of the Jak/STAT3 pathway led to increased GFAP expression, while the IL-6-R-Ab administration reduced astrogliosis.

Small-fiber neuropathy is frequently described in fibromyalgia [104]. It refers to the selective loss of unmyelinated C and thinly myelinated Aδ fibers, which mediate pain, heat and cold sensation, respectively. Small-fiber neuropathy is classically slowly progressive and length-dependent in onset, although non-length-dependent forms exist. Therefore, the identification of a small fiber or early sensory neuropathy in the setting of widespread pain is important and carries clinical-management implications [105]. In this scenario, another crucial point is the overexpression of proinflammatory mediators, which are responsible for central sensitization during the development of pathologic pain [34]. In particular, when the STAT3 pathway is activated, reactive astrocytes become important sources of chemokines [106]. Our data showed the upregulation of Cxcl10, Cx3cl1 and Cxcl5 in the dorsal horns of the spinal cords of the suffering animals, while the IL-6-R-Ab administration significantly reduced their expression. Chemokines from reactive astrocytes induce multiple effects on neuroinflammation. It was reported that they are able to regulate the expression of proinflammatory genes [107], increasing nociceptive transmission through activated microglial cells [108]. Firmly in line with the literature, the increase in chemokines in the animals with fibromyalgia was correlated with increased Iba1 expression, while the IL-6-R-Ab administration significantly reduced microglial activation.

Various pathological-pain models showed increased expression levels of interleukin-6 and its receptor in the spinal cords and dorsal root ganglia. In summary, our study showed that IL-6 has a key role in fibromyalgia, activating the Jak/STAT3 pathway, chemokine overexpression and glial-cell activation. Our data suggest that the modulation of the IL-6 pathway could attenuate pain-like behavior in fibromyalgia-dependent pain. The findings from our preclinical study may have clinical implications for enhancing our understanding of the molecular mechanisms underlying the disease and identifying potential new targets for therapy.

## Data Availability

The data used to support the findings of this study are available from the corresponding author upon request.
